# Institutional Diversity in Response to the COVID-19 Emergency

**DOI:** 10.3390/healthcare8040515

**Published:** 2020-11-27

**Authors:** Jingjing Yan, Dahai Zhao

**Affiliations:** 1 School of International and Public Affairs, Shanghai Jiao Tong University, Shanghai 200030, China; yanjingjing@sjtu.edu.cn; 2 Shanghai Jiao Tong University-Yale Joint Center for Health Policy, Shanghai 200030, China

**Keywords:** institutional diversity, COVID-19 emergency response, four-quadrant model

## Abstract

Four-quadrant modeling may offer some constructive insights into the institutional diversity of the emergency responses to COVID-19. This study utilized a typological method to investigate institutional arrangements and the emergency management of epidemic responses in China. The task environment for emergencies was divided into four categories. Targeted policies were assigned to explicit task environments by placing them in one of the four quadrants: public health procedures, medical operation standards, supervisory and regulatory measures, and norms and instructions. Institutional diversity resulted when the very loci of decision-making carried a dominant or subordinate role, providing a hierarchical system for relating the institutional processes needed to address the challenges of institutional fragments.

## 1. Introduction

Sweeping through the globe in 2020, COVID-19 continues to have a huge impact in many countries across the world. As part of the emergency response, the governments of major COVID-19-endemic countries have introduced measures to contain the spread of the infection and protect their populations. As the events surrounding the spread of the disease continue to unfold, we have seen similar measures producing different results across countries [1]. For example, stringent public health procedures have proven to play a crucial role in avoiding substantial increases in both the cases and fatalities of COVID-19; this is particularly true in cases where the virus transmission route is unclear and the entire population is susceptible [2]. However, while the implementation of social distancing regulations, quarantine, and enforced periods of isolation have proven to be effective in containing the spread in some countries, other regions have encountered resistance from the people. Indeed, when institutional measures and governing regulations are implemented in an immoderate, heavy-handed, or haphazard manner, they can lead to skepticism and the infringement of human rights, with potentially threatening consequences [3]. Regarding the deep uncertainty and profound vulnerability of emergency responses, in this study, a set of institutional measures was compared in the literature in light of the current context.

A number of studies have evaluated specific public health measures and their corresponding performance in a particular place or region with respect to COVID-19 [4,5,6,7]. Some studies have drawn comparative conclusions regarding bureaucratic systems, as specific experiences and information pertaining to the public health sector have recently been obtained [8,9]. As much of the literature focuses on governmental issues—or on expectations regarding the outcomes of institutions in terms of the innovativeness of public health emergency management—there is a distinct lack of studies concerning the strategy of sequential updating and institutional structuring in response to a pandemic.

Nevertheless, in the literature on institutional and organizational theories, institutional diversity is regarded as a prerequisite for organizing measures to realize government orders [10,11]. For example, in the higher education sector, institutional diversity explains the interaction of complex factors that drive educational institutions to meet the needs of diverse groups [12,13]. In the field of international relations, institutional diversity constitutes a framework within the system of international law [14,15]. In the marine sector, institutional diversity harmonizes the global principles of activities and protects marine biodiversity and sustainable development [16,17]. The notion of institutional diversity further addresses the issues surrounding risk management and profit distribution in the agricultural sector [18,19]. This implies that leading modes of governance and administration are established based on the rationales of institutional diversity, which initiates specific practices with significant outcomes. In this sense, institutional diversity is regarded as a carrier whose function is derived from the dynamics and interactions of institutions, which in turn act on the construction of institutions to form a certain order [20,21,22,23]. In this way, the notion of institutional diversity can be proposed as a means to understand the diverse institutions present in responding to COVID-19 and can be applied extensively in the field of public health emergency management.

Consequently, this study analyzes such institutions in China’s response to COVID-19 from the perspective of institutional diversity in public emergency management. The study also proposes a specific model as the dominant framework in which the emergency responses take place. The study focuses on China as the country experienced the pandemic first and used a heavier first-touch response than other states; furthermore, the Chinese government has been relatively active in terms of its institutional contribution. This manuscript aims to contribute to academic and practical circles in two ways. First, it proposes a practical model of institutional structuring regarding a country’s response to emergencies by allowing the implementation of institutional design in public health emergency management. Following this model, policymakers in other countries may be better prepared to design their own state’s responses to emergencies. Second, a concrete and reproducible institutional evaluation tool is proposed (based on practical innovations), which can produce more cases and evidence for the use of theories of institutional diversity, as gleaned from altered research areas. For this purpose, we use the model of institutional diversity and discuss aspects of both institutional structuring and political compromise in providing the emergency response to the COVID-19 pandemic [24,25,26]. The model is employed to highlight relationships among different institutions, with varied functions, which maintains the institutional design of decision-makers and their schemata for resource use, while also linking these schemata and institutions on a larger scale [27].

## 2. Methods and Materials

This study utilized a typological method to investigate the classification of institutions. To begin with, it refined existing conceptualizations by recognizing “complexity”, “variety”, “instability”, and “unpredictability” as four environmental elements. These elements, in turn, represent the environmental tasks and consequences present in the wake of a pandemic. In view of these ideas, a typology that identifies the four categories of institutional measures and responds significantly to environmental elements was introduced. Furthermore, institutional diversity was considered by proposing the features of the interactions between each environmental element and the explicit institutional measure. In view of this typology, the strategies of institutional diversity present in the responses to COVID-19 were determined. All cases discussed in this manuscript were taken from the literature, government sources, and mainstream media websites.

Referring to the research materials, we obtained peer-review literature from Google Scholar through the keywords of “COVID-19, policy” or “COVID-19, response”. To construct the theoretical framework of the manuscript, we further collected some peer-review literature, dissertations, and books on emergency response, public health management, and organization theories. The research materials involved were in English. Referring to the case study, we collected policy texts related to COVID-19 response on the websites of the central ministries and commissions of China from December 2019 to April 2020 by manual retrieval. The policy measures were mainly launched during this phase, a major period of the epidemic. The criteria for policy selection were based on the theoretical framework of the manuscript. The policy texts in this section were publicly available information in Chinese. In addition, we also obtained data and case reports on the websites of International Monetary Fund (IMF) and Johns Hopkins Coronavirus Resource Center, among others, through fixed-point retrieval. This part was publicly available information in English. The retrieval links for all of the above information and the last retrieval time for this manuscript were indicated in the citation.

## 3. Conceptualizations of Environmental Elements in COVID-19 Emergency Responses

Unlike procedures and solutions that were applied to manage experience-based tasks, policy measures employed for the emergency response to COVID-19 were taken without ascertaining whether they could advance steadily and consistently. In other words, we did not foresee the outcomes, as the challenge was still new and ongoing. In this manner, the use of a typological method gave us a starting point by specifying the environmental tasks in relation to the COVID-19 emergency. Here, we referred to the discourse from Shortell et al. [28,29,30,31,32,33,34,35,36], as we believed that the following propositions were applicable in analyzing the institutional surroundings in the emergency response to COVID-19. Specifically, we used environmental elements referring to a certain type of environmental task.

Briefly, for the government, the outbreak and spread of COVID-19 triggered not only healthcare-related tasks, but also general management-related tasks regarding the emergency response. All tasks changed rapidly as the infection progressed and in light of greater knowledge of the virus obtained by both the Ministry of Health and the respective administrative departments. The changing dynamics of healthcare-related tasks were the result of different symptoms, immunities, and the day-to-day interactions of human communities, while the changing dynamics of general management-related tasks resulted in ever-growing political, economic, and social affairs crises—thus expressing a future view. Therefore, in terms of both tasks, as well as their changing status, the four categories of environmental elements were identified.

### 3.1. Environmental Complexity

According to Shortell et al., complexity refers to the environmental tasks that must be accounted for [28]. Environmental complexity is referred to as healthcare-related tasks directly arising from the pandemic. Since the outbreak was reported in Wuhan at the end of 2019, the number of COVID-19 cases has grown rapidly. Shortly after the outbreak was reported, cases expanded beyond Wuhan to the rest of the Hubei Province, as well as many other cities across the country. In a day or two, the country had accumulated an incredibly large number of COVID-19 cases, which promptly triggered healthcare-related tasks [37]. These tasks pertained to the control of the spread of the infection and further prevention of COVID-19-related deaths. The tasks were promptly carried out across different locations; however, the need for a response was identified in so many different gatherings of individuals over a short period of time that we defined it as being emblematic of the environmental complexity of the pandemic.

### 3.2. Environmental Variety

Variety refers to the degree to which tasks differ from one another [28]. Environmental variety is referred to as the general mix of management-related tasks present in the emergency response to COVID-19, apart from the healthcare-related tasks. For instance, this pandemic has precipitated economic crises and social inequalities, which has resulted in worldwide unemployment, monetary downturns, and fiscal strain (among others)—broadly categorized as concerns other than the loss of life. As reported, the pandemic has caused 14 million job losses in the United States alone through April 2020 and affected more than 80 percent of the global workforce. The global economy was expected to shrink by 3% in 2020, a much steeper decline than that experienced throughout the 2008/2009 financial crisis [38]. These general management-related tasks showed certain objective distinctions from the healthcare-related tasks.

### 3.3. Environmental Instability

Instability refers to the rapidity with which tasks change over time [28]. Environmental instability describes the attributes of the dynamic status of healthcare-related tasks. In the response to COVID-19, healthcare-related tasks were changing as a result of factors such as the mutation of the virus, fluctuations in the immunity of the population, and advances in medical techniques for treating the infection. Thus, we refer to environmental instability as the developing nature and probable outcomes of the changes in healthcare-related tasks. For example, the symptoms of COVID-19 have varied according to underlying diseases present in individual cases [39]. Age also plays a major role in the fluctuating severity of symptoms. Several countries have claimed the development of, and the start of clinical phases pertaining to, a COVID-19 vaccine. However, progress relating to vaccine research has been erratic [40]. The healthcare-related tasks were, therefore, unstable over time. While the task showed the dynamic features, we defined it as the environmental instability of the pandemic.

### 3.4. Environmental Unpredictability

Unpredictability refers to the extent to which tasks can be predicted [28]. Environmental unpredictability describes the changes in general management-related tasks over time. Considering the scope of general management-related tasks, the COVID-19 pandemic caused massive, and still unknowable, suffering across a wider scope. This unknowable nature of the pandemic has required unprecedented and unpredictable tasks from the government. The general management-related tasks influenced many departments, and the probable outcomes were unlikely to be easy to address. For example, it is still not clear how long the threat will last and what effects it will have on global wellbeing, economy, and social issues; tremendous losses regarding these aspects have already been incurred since the outbreak [41]. The difference between environmental unpredictability and environmental instability is that the prevailing unpredictability emphasizes that the scope of change is not limited to homogeneous outcomes—outcomes that involve changes to a wide range of factors other than those related to healthcare.

## 4. Refined Typologies of Institutions in COVID-19 Emergency Responses

### 4.1. Public Health Procedures for Environmental Complexity

**Figure 1:**
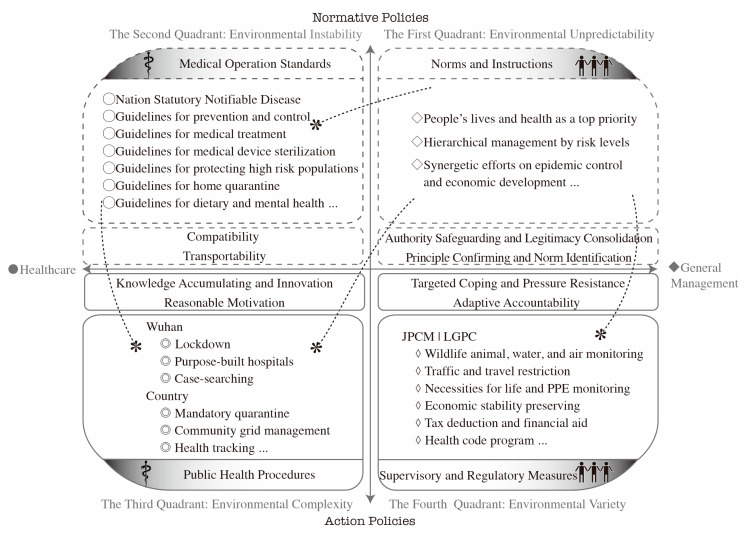
Institutional diversity in COVID-19 emergency response. The first quadrant concerns norms and instructions with respect to environmental unpredictability. The second quadrant concerns medical operation standards with respect to environmental instability. The third quadrant pertains to public health procedures with respect to environmental complexity. The fourth quadrant regards supervisory and regulatory measures, with respect to environmental variety. The policies and strategies of each quadrant are described in the following sections of the text. The direction of the dotted arrows indicates that one group of policies is conditional on the other. JPCM: Joint Prevention and Control Mechanism of the status Council, LGPC: Leading Group of the CPC Central Committee on the Prevention and Control of the COVID-19, PPE: personnel protective equipment.

Whether because of the rapidly increasing number of infected cases or the spread of infected areas, a set of healthcare-related measures was urgently needed to control the spread of the infection and to treat those with COVID-19. Public health procedures were subsequently organized to control healthcare-related tasks. For example, soon after Wuhan made nine announcements regarding its lockdown on 21 January 2020, two purpose-built hospitals (Leishenshan and Huoshenshan) and Fangcang shelter hospitals were dedicated to receiving COVID-19 patients [42]. In the following week, Wuhan issued the tenth announcement to expand case searches in the city, now covering patients with fevers and pneumonia, while also identifying the close contacts of positive cases to avoid further transmission [43]. Since this search, the number of inpatients in Wuhan promptly skyrocketed, which instantaneously overloaded Wuhan’s already stretched medical systems (at this point, hospitals and medical facilities were faced with over 60% of the country’s patients). Under these circumstances, Wuhan set up an innovative purpose-built hospital (Fangcang shelter hospital) to treat those with mild symptoms and asymptomatic infections, ensuring that as many people with COVID-19 as possible could receive free medical observation and treatments by the government [44]. In the rest of the country, a mandatory 14-day home quarantine for medical observation was also imposed for those who had recently traveled to Wuhan. Some economically developed provinces imposed local measures for further search and to offer help to suspected cases within their cities; for example, an online guidance center was established in Fujian province and a 24 h online platform for healthcare services was introduced in Zhejiang province [45]. Expanded case searching, grid monitoring, and the comprehensive management of communities became the most important procedures for public health in terms of controlling the pandemic. An additional 14-day isolation was declared for individuals who were discharged from the medical center, while the search for contagions was further expanded to tracing those who had purchased drugs to combat fever as well as to frequently traveled routes intersecting with high-risk places after 20 January. This is shown in Figure 1.

### 4.2. Supervisory and Regulatory Measures for Environmental Variety

In terms of environmental variety, supervisory and regulatory measures (with regulatory functions for public affairs) have been taken from a range of comprehensive and broad viewpoints—namely, measures organized to deal with the general management-related tasks, as shown in Figure 1. Remarkably, the Joint Prevention and Control Mechanism of the State Council (JPCM) and the Leading Group of the CPC Central Committee on the Prevention and Control of COVID-19 (LGPC)—headed by Premier Li—set up a decision-making exchange platform consisting of multiple ministries and governments, at various levels, for an exhaustive reaction to the pandemic [46]. During the domestic outbreak, supervisory and regulatory measures exerted by the JPCM were executed across a diverse range of domains—such as the monitoring of wildlife to identify intermediate hosts, air and water monitoring for safe survival, implementation of traffic and travel restrictions to control the spread, supply of daily necessities and personnel protective equipment (PPE), provision of psychological first aid, provision of child and elderly care, implementing tax deductions and providing financial help, punishing instances of price and quality cheating in medicine, and donation and supply auditing. The LGPC arranged job preservation schemes, greater medical supply production, deployment of medical workers, development of drugs and vaccines, and anti-epidemic work in key infected areas of Wuhan. After the domestic outbreak was basically under control, the JPCM administered an economic resumption package for small enterprises and financial aid for the population and economic units affected by the pandemic. After foreign imports began to dominate the situation, the JPCM organized international cooperation on pandemic prevention and control. At the domestic level, a citizen health code program was promoted for public health security management on a regular basis, with each individual’s health risk information indicated and updated by means of their phones via a colored QR code (used when accessing public places) [37].

### 4.3. Medical Operation Standards for Environmental Instability

In terms of environmental instability, a series of medical standards were set up to ensure that lives could be saved and persons with COVID-19 could be treated in a regularized, clinical way. These medical operation standards were organized when facing healthcare-related tasks, as shown in Figure 1. COVID-19 was first listed as a national statutory notifiable disease [47]. Following the national standards for infectious disease, the prevention and control guidelines, medical treatment guidelines, plasma treatment guidelines, traditional Chinese medicine treatment guidelines, rehabilitation guidelines, discharged patient management guidelines, and psychological aid guidelines were put into practice. According to the preparedness principle—based on the different risk levels—several plans for protecting high-risk populations, places, and communities were also put in place. In addition, for the economical and rational use of medical equipment, face mask guidelines, unified design criteria for medical emergency facilities, and medical device sterilization were developed. Additionally, daily life guidelines were provided, such as dietary guidelines for pneumonia prevention, mental health guidelines for pneumonia prevention, and home quarantine and self-isolation guidelines for medical observation. Furthermore, medical operation guidelines for clinical therapy and research were emphasized once information on understanding and treating COVID-19 was accumulated.

### 4.4. Norms and Instructions for Environmental Unpredictability

In terms of environmental unpredictability, the pandemic continued to cause massive (and still unknowable) suffering on a wider scale and the circumstances remained unpredictable. The instructions concerning the directing value and principles for managing an unpredictable emergency were laid out, namely, the clarification of norms and instructions for times when faced with the need for general management-related tasks, as shown in Figure 1. During the rapid outbreak, widespread panic and rumors spread parallel to the number of diagnosed cases, posing threats to centralized authoritativeness and legitimate leadership. Bearing this in mind, one fundamental instruction was to spare no effort in controlling the pandemic and to save lives. Protecting the health of the entirety of the population was considered as the most elevated principle for the medical community [48]. Given this, legitimacy was accurately positioned with mass-based interests in the emergency response, and the reinforcement of a centralized administration system, chaired by the COVID-19 containing headquarters (JPCM and LGPC), furthered these efforts. In terms of pandemic prevention and control, COVID-19 containment in Wuhan was of utmost importance. With a deficiency in medical equipment and hospital admission capacity, hierarchical management based on symptoms was incorporated into the disseminated instructions. Furthermore, the issue of economic preservation also received tremendous concern from the central authorities. In the first quarter, a number of small- and medium-sized enterprises went bankrupt, while surviving companies laid off employees or reduced salaries. Nevertheless, the impact on the economy partly depended on the public’s reaction to the disease [49]. The COVID-19 containment headquarters have started to coordinate economic development in a similar way to the initial epidemic response.

## 5. The Four-Quadrant Model of Institutional Diversity

As shown in Figure 1, institutional measures responding to the emergency were additionally analyzed in a particular framework, as shaped by the intersection of vertical and horizontal axis, depending on environmental elements and corresponding institutions. By developing a horizontal and vertical axis framework, the institutional diversity model was framed along with four quadrants that were contingent upon whether the institutional measures were normative or action-based, and whether the measure type concerned healthcare or general management. This indicated that an institutional system used in response to an epidemic was formed by the endeavors of the four explicit methods.

The vertical axis represents normative and action policies. Action policies were targeted actions for specific tasks. As analyzed above, environmental complexity spoke to healthcare-related tasks regarding the situation concerning the COVID-19 emergency response. Specific healthcare-related tasks required targeted public health actions. These actions included the implementation of lockdowns, the creation of purpose-built hospitals, case searching in Wuhan, enforcing mandatory quarantine, community grid management, and the health tracking of the country. The environmental variety implied that, notwithstanding healthcare-related tasks, there were different kinds of general management-related tasks regarding emergency response. A single mode of public health action cannot adapt to the general range of tasks, and extensive modes of action are expected, such as the monitoring of wildlife, water, and air; implementation of traffic and travel restrictions; provision of necessities for life and PPE monitoring; economic stability preservation; introducing tax deductions and financial aid; providing health code programs; and other supervisory and regulatory measures as taken by the JPCM and LGPC. To summarize, action policies focused on targeted and specific acting modes and patterns, which planned explicit acts to manage the complexity and diversity of the environment and which are pertinent to the tasks at hand [50,51].

Normative policies are a set of long-term goals and values that indirectly support actions. In other words, normative policies did not provide practical actions for a particular environmental task, and the significance of normative policies lays more in setting goals and values for governing different behavioral patterns. This was because of environmental instability and given that the circumstances of healthcare-related tasks may change quickly in the future, thus presenting an evolving dynamic. At this point, as a particular healthcare-related task changed, the former action-based measure in reaction to this task was no longer fully applicable. Thus, a principle has been proposed that will allow one to respond to the different status of a particular healthcare-related task when the circumstances of the task fluctuate. This refers to the medical operation standards, such as guidelines for COVID-19 prevention and control and for the medical treatment, which have offered very specific healthcare-related actions with a set of principles to ensure that each individual is treated with a national standard, recognized by the codes and laws related to the National Notifiable Diseases Surveillance System, despite their individualized differences. In addition, because of environmental unpredictability, changes in general management-related tasks are often difficult to foresee and dissect through experiences or theories in a complete and accurate manner. In this way, normative policies for general management-related tasks have set common values and headings in advance. This refers to the norms and instructions, the idea that people’s lives and health are a top priority, practicing hierarchical management by risk levels, promoting synergetic efforts in epidemic control, and economic development. For a situation in which it is difficult to set complete and total action-based responses, preset scenarios restrict all-action modes and patterns to the established value and headings. It was argued that normative policies played a supporting role in the aftereffects of new tasks.

Moreover, the policy types of healthcare and general management (as they appear along the horizontal axis) show that there have been applicable action policies (or normative policies) for healthcare, as well as other extensive action policies (or normative policies) for general management of the emergency response to COVID-19. To sum up, in an effort to tackle the epidemic, the norms and instructions, a group of normative policies for general management, uphold the other three policy categories in terms of goals and values owing to their extensive content and far-reaching principles. Medical operation standards, a group of normative policy of healthcare, comprised a group of standards and guidelines in the healthcare category that indirectly guided the action policies of healthcare. Public health procedures, the action policies of healthcare, were explicit actions and procedures enacted to deal with explicit healthcare problems. Supervisory and regulatory measures, that is, the action policies of general management, were specific action measures that deal with the extensive problems of non-healthcare-related aspects. Through these four categories of institutions, institutional diversity (with the logic of hierarchical correlation) has been framed around China’s joint prevention and control activities in response to COVID-19.

## 6. Strategies of the Four-Quadrant Model of Institutional Diversity

### 6.1. Authority Safeguarding and Legitimacy Consolidation

In the first quadrant, there was a group of norms and instructions. The main strategy in the emergency response of policies located in the first quadrant was authority safeguarding and legitimacy consolidation. The major norm and instruction in this emergency response was a high priority for people’s wellbeing. In this manner, all approaches were introduced to protect lives, such that all issued policies stood for the interests of the vast majority. This, in turn, helped strengthen the legitimacy of the rest of the policies by creating a solid foundation. In addition, much of the Chinese population was further affected financially during the outbreak. As financial impacts and losses were irreparable, the norm and instruction regarding economic reconstruction guaranteed the eventual fate of predictable growth and prosperity when economic losses are incurred in the fight against the epidemic. As the government offered prompt responses to the economic sector alongside the public emergency, norms and instructions were regularly developed to consolidate the legitimacy of governance as a process of authority safeguarding [52].

### 6.2. Principle Confirming and Norm Identification

The other strategy in responding to emergencies through policies, as found in the first quadrant, was principle confirming and norm identification. The immediate consequence of authority safeguarding was to give all principles and norms a sense of logical correctness, which can receive mass recognition. Such recognition made action policies seek mutual identity spontaneously. This was based on the view that the ideas and ideals of norms and instructions were confirmed when policies were issued. For example, “people’s lives and health as a top priority” sent a strong message to the country that government departments should do all that they can to save lives. When such a policy was issued, the conviction was confirmed within other norms and acts. These safeguards also brought progressive adjustments to new instructions within a changing situation, altering existing instructions and generating complementarity. With principle confirming and norm identification, the goals and values conveyed by the norms and instructions were updated.

### 6.3. Compatibility

The main strategy in the second quadrant was compatibility between policies. The normative policy provided a certain goal and direction by guiding action policies, as all policies pinpointed the fixed principles. The conflicts endured during the implementation or interpretation of action policies will be readjusted according to established principles. For example, in the early stage of the epidemic, the number of face masks acquired fell far short owing to the growing demand for surgical care. Some proposals to recycle masks have been widely circulated, such as washing masks with water and spraying and disinfecting masks with alcohol. However, some measures denied reusing masks and pointed out that the effective layer of the masks is destroyed and loses its protective function after washing. In response to the different views of self-protection, the government issued guidelines for medical device sterilization in a timely manner. Thus, action policies specifically kept inherent consistency and appeared to be mutually compatible [53]. This was reflected, in particular, in medical operation standards. Professional operation standards in medical care represented the rules required in medical operations, which were compatible owing to the professionalism achieved by the continuous updating of each medical operation standard.

### 6.4. Transportability

Another strategy in the second quadrant was the transportability of policies. Transportability is generated by interactions between health experts and policymakers. Normative policies of medical operation standards were practiced at each level, along with the reputation of medical authorities (such as Nanshan Zhong, a well-known national health specialist). The goals and values of healthcare professionals were transferred through health departments and implemented by local administrative departments on the same level. The intensive cooperation between health and administrative departments guaranteed that medical operations were practiced using standardized approaches across different levels and locations. This led to policy transportability across hierarchical levels, such as self-quarantining and discharge management, which was implemented with the cooperation and assistance of administrative and law enforcement departments, in accordance with medical operation standards. According to the principles of medical operation standards, there are very clear references for the implementation of specific healthcare measures. Although the conditions for implementing self-quarantine and discharge management were different at different governmental levels, the second quadrant policies transmitted a set of transplantable operating standards in each health initiative, in order to ensure effectiveness.

### 6.5. Knowledge Accumulating and Innovation

The third quadrant contained a group of public health procedures. The main strategy in this quadrant was knowledge accumulation and innovation. COVID-19 was not characterized as resembling any previously known coronavirus. This novel coronavirus infection caused a large number of both mild and asymptomatic infections, which exacerbated the spread of the disease. The social activities of the mildly symptomatic and self-isolated population were evidently contributing to its exacerbation. Therefore, stadiums and exhibition centers were reformed into large-scale, temporary hospitals that provided medical monitoring and accommodation, as well as social interaction activities for isolated populations with mild to moderate infection. The Fangcang shelter was built on a military field hospital, converted to novel healthcare facilities during the emergency response [54]. This helped to interrupt community spreading and became one of the most impressive measures in the response to COVID-19. Innovations and knowledge from these public health procedures have been accumulated for future emergency responses.

### 6.6. Reasonable Motivation

The other strategy in the third quadrant was reasonable motivation. Some measures were set up while providing reasonable motivation to front-line medical workers and epidemic prevention staff members at the grassroots level. Community keepers experienced this sense of motivation while safeguarding their communities and responsibility for outside-to-home services during the period of social restrictions. For example, Shanghai’s community epidemic prevention was outstanding on a national scale, especially as the majority of doctors in Shanghai were assigned to serve in Wuhan. This success in Shanghai was due to “community aunts” in community grid management, who were safeguarding their communities as gatekeepers. Action policy was a reasonable motivation, owing to the innovativeness of its process. When a policy is engaged in continuous social action, it provides progressively more proficient solutions to existing issues. During this process, knowledge was rapidly adopted and accumulated into existing arrangements, with more motivated choices updated in order to deliver productive outcomes.

### 6.7. Targeted Coping and Pressure Resistance

The main strategy in the fourth quadrant was targeted for coping and pressure resistance. When confronted with non-routine tasks, action policies were allocated as countermeasures to resist stress and tackle specific issues. For example, the Department of Ecological Protection tested the water source and air to ensure cleanliness. Restrictions on transport and tourism were also enforced to prevent the spread of the epidemic in cases of cluster infections. Wearing face masks has become a typical practice in the prevention and control of the epidemic. However, the number of masks and other medical resources in China, in the beginning, was far below what was necessary for epidemic prevention. Subsequently, state-owned enterprises launched plans to guarantee the production and circulation of medical supplies. These measures were unintended target-coping reactions to the sudden, uncontrollable spread of COVID-19. In this manner, the action policy was issued for targeted coping and pressure resistance, which generated certain solutions to specific task elements.

### 6.8. Adaptive Accountability

The other strategy in the emergency response of policies in the fourth quadrant was adaptive accountability. As the point of action policy was merely task tackling, it acknowledged what happened and adapted to the changing task, but ignored other aspects. These aspects usually caused a negative social impact or social loss. Action policy caused hysteresis in public emergency response while practicing target coping, which meant that it addressed the problems that had emerged in order to implement countermeasures. In this sense, action policy rejected universal accountability, but built adaptive accountability. Meanwhile, it can be seen that stringent measures were taken for COVID-19 containment so as to rectify the governing order. This shows that the action policy was good at bias correction. This originated out of procedures of targeted coping, which, in turn, formed a unique accountability and supervision process.

## 7. Limitations

This study has some limitations. Shortell et al. expanded environmental elements to hostility and dependence in 1977. However, the current study only lists the four most prevalent categories of environmental elements according to the logic that echoed the policy categories discussed. This begs the question of, which came first, the chicken or the egg? Did the division of environmental elements precede the division of institutions? This manuscript argues that institutions were designed according to the task environment in response to the emergency. Thus, the most representative environmental elements were presented in conceptual form as a basis for analyzing diverse institutions. Therefore, academic discourse on other categories of differentiation is needed in the future.

## 8. Conclusions

From the above analysis, it can be seen that, in the response to major health emergencies, a lot of emphasis is placed on the application of the combinations between different policy measures. To be specific, governance objectives in different aspects should be formulated according to different environmental tasks, and different response policies should be applied to these governance objectives. Finally, the combined effects of policies can be achieved through the effective identification of the task environment and the reasonable setting of policy measures and programs. In this respect, the policy combination of the four quadrants proposed in this manuscript presents the practical case. Besides, an important assumption of the manuscript was that the institutional surroundings and time were variables. As key evidence and information were going to being revealed in the course of the pandemic, specific action policies should be altered to deal with the emerging tasks in the third and fourth quadrants. That is, the key principles and goals for major health emergency management in the first and second quadrants were pre-established to be crucial until more explicit actions were set in the third and fourth quadrants.

New policies for COVID-19 containment are still being introduced, and the logic of the four-quadrant modeling of institutional diversity to the COVID-19 response has become clearer in policy updates. Although the model itself is not an explicit guide for institutional settings, this study found that the process of the four-quadrant modeling renewal produces a functional structure. In terms of institution structuring, hierarchies were represented in the four-quadrant framework, which is related to structures based on the top-down delegation of power. This meant that some institutions were subject to domination by others. Although acknowledging this fact implies the importance of focusing on the predominant relationship of institutions [55], the hierarchy of institutions organized them into a framework whereby the very loci of decision-making carried either a dominant or subordinate role. In a healthcare or general management method, they provided a system for relating the institutional process to address the challenges of institution fragments [56,57,58,59]. Institutional diversity, through the formation of complementary and dynamic frameworks and relationships, applies the prevailing governance model and rationale to the practice of public health emergency management, thereby creating a unique order to combat the epidemic.

## References

[1] Turner M.J., Oluwatoyin A., Layschel K. (2020). Examining Social Determinants of Health, Stigma, and COVID-19 Disparities. Healthcare.

[2] The Lancet (2020). COVID-19: Too Little, Too Late?. Lancet.

[3] Parmet W., Michael S. (2020). Covid-19—The Law and Limits of Quarantine. N. Engl. J. Med..

[4] Haskell W., Steven B., James H. (2019). Physical Activity: Health Outcomes and Importance for Public Health Policy. Prev. Med..

[5] Vicente N., Carles M., Carme B., Joan B., Áágueda Q., Maica R., Núria V. (2006). Politics and Health Outcomes. Lancet.

[6] Elizabeth B., Maureen C., Erika R., Talbert-Slagle K., Ndumele C., Taylor L., Curry L.A. (2016). Variation in Health Outcomes: The Role of Spending on Social Services, Public Health, and Health Care. Health Aff..

[7] Smith K., Bambra C., Joyce K., Perkins N., Hunter D.J., Blenkinsopp E.A. (2009). Partners in health? A systematic Review of the Impact of Organizational Partnerships on Public Health Outcomes in England between 1997 and 2008. J. Public Health.

[8] Jinrui Z., Ruilian Z. (2020). COVID-19 in China: Power, Transparency and Governance in Public Health Crisis. Healthcare.

[9] Kaifeng Y. (2020). Unprecedented Challenges, Familiar Paradoxes: COVID-19 and Governance in a New Normal State of Risks. Public Adm. Rev..

[10] Boyer R. (2004). New Growth Regimes, But Still Institutional Diversity. Socio-Econ. Rev..

[11] Daft R. (1998). Essentials of Organization Theory and Design.

[12] Núñez A., Crisp G., Elizondo D. (2016). Mapping Hispanic-Serving Institutions: A typology of institutional diversity. J. Higher Educ..

[13] Morphew C. (2009). Conceptualizing Change in the Institutional Diversity of US Colleges and Universities. J. High. Educ..

[14] Seibel H. (2008). Islamic microfinance in Indonesia: The Challenge of Institutional Diversity, Regulation, and Supervision. J. Soc. Issues Southeast Asia.

[15] Morgan G. (2009). Globalization, Multinationals and Institutional Diversity. Econ. Soc..

[16] Jones P., Qiu W., Santo E. (2013). Governing Marine Protected Areas: Social–ecological Resilience through Institutional Diversity. Mar. Policy.

[17] Rayner S. (1999). Mapping Institutional Diversity for Implementing the Lisbon principles. Ecol. Econ..

[18] Michelsen J. (2002). Organic Farming Development in Europe—Impacts of Regulation and Institutional Diversity. Economics of Pesticides, Sustainable Food Production, and Organic Food Markets.

[19] Tschirley L. (2010). Institutional Diversity and Performance in African Cotton Sectors. Dev. Policy Rev..

[20] Hiroshi N. (2010). Institutional Hierarchy Hypothesis, Multilayered Adjustment, and Macroeconomic Performance: A Post-Keynesian Dynamic Approach. Evol. Inst. Econ. Rev..

[21] Hodgson G. (1993). Economics and Evolution: Bringing Life Back into Economics.

[22] Robert B. (2005). Coherence, Diversity, and the Evolution of Capitalisms—The Institutional Complementarity Hypothesis. Evol. Inst. Econ. Rev..

[23] William W. (2005). Hierarchy Theory in Sociology, Ecology, and Resource Management: A Conceptual Model for Natural Resource or Environmental Sociology and Socioecological Systems. Soc. Nat. Resour..

[24] Paul A. (2001). Market, Hierarchy, and Trust: The Knowledge Economy and the Future of Capitalism. Organ. Sci..

[25] Eric M. (2002). Path-dependence and Initial Conditions in the Transition Process: The Cases of Hungary and Romania. J. Econ. Bus..

[26] Hall P. (2005). Institutional Complementarity: Causes and Effects. Socio-Econ. Rev..

[27] Ahrens J., Joachim P. Transitional Institutions, Institutional Complementarities and Economic Performance in China: A ‘Varieties of Capitalism’ Approach (Working Paper).

[28] Shortell S. (1977). The Role of Environment in a Configurational Theory of Organizations. Hum. Relat..

[29] Osborn R., Hunt J. (1974). Environment and Organizational Effectiveness. Adm. Sci. Q..

[30] Cilliers P. (2005). Complexity, Deconstruction and Relativism. Theory Cult. Soc..

[31] Cilliers P. (2006). On the Importance of a Certain Slowness. Emerg. Complex. Organ..

[32] Dill W. (1958). Environment as an Influence on Managerial Autonomy. Adm. Sci. Q..

[33] Thompson J. (1967). Organizations in Action.

[34] Emery F., Trist E. (1965). The Causal Texture of Organizational Environment. Hum. Relat..

[35] Child J. (1972). Organization Structure and Strategies of Control: A Replication of the Aston Study. Adm. Sci. Q..

[36] Perrow C. (1967). A Framework for the Comparative Analysis of Organization. Am. Sociol. Rev..

[37] Jingjing Y., Dahai Z. (2020). Administrative Mechanism of Joint Participation and Cooperation in the Early Stages of the COVID-19 Outbreak in Wuhan. Risk Manag. Healthc. Policy.

[38] International Monetary Fund (IMF) (2020). Chapter 1. Policies to Support People During the COVID-19 Pandemic. Fiscal Monitor, April 2020.

[39] Robert V., Lucy O., Dorigatti I., Winskill P., Whittaker C., Imai N., Cuomo-Dannenburg G., Thompson H., Walker P.G.T., Fu H. (2020). Estimates of the Severity of Coronavirus Disease 2019: A Model-based Analysis. Lancet Infect. Dis..

[40] CBSnews Oxford’s COVID Vaccine Trial “Back Up and Running” after Brief Pause for Safety Check. https://www.cbsnews.com/news/covid-vaccine-oxford-trial-astrazenca-resumes-safety-check/.

[41] Johns Hopkins Coronavirus Resource Center COVID-19 Map (17 April 2020). https://coronavirus.jhu.edu/map.html.

[42] Wuhan Official Wuhan Will Build Another “Xiaotangshan Hospital” with 1300 New Beds. 25 January 2020. https://weibo.com/2759348142/Ir8vf9QwK.

[43] Wuhan Municipal Government Municipal Headquarters for the Prevention and Control of Novel Coronavirus Pneumonia (Announcement no. 10). 2 February 2020. http://www.hubei.gov.cn/zhuanti/2020/gzxxgzbd/zxtb/202002/t20200202_2018011.shtml.

[44] Wuhan Municipal Government Wuhan International Convention and Exhibition Center Will Be Changed to “Fangcang Shelter Hospital”. 4 February 2020. http://www.hubei.gov.cn/zhuanti/2020/gzxxgzbd/zxtb/202002/t20200204_2018745.shtml.

[45] State Council Information Office Fujian Holds the 14th Press Conference on Joint Prevention and Control of COVID-19 Epidemic. 18 March 2020. http://www.scio.gov.cn/xwfbh/gssxwfbh/xwfbh/fujian/Document/1675590/1675590.htm.

[46] National Health Commission The National Health Commission and the Relevant Departments Jointly Prevent and Control the Pneumonia Epidemic of New Coronavirus Infection. 21 January 2020. http://www.nhc.gov.cn/xcs/fkdt/202001/d9570f3a52614113ae0093df51509684.shtml.

[47] National Health Commission Novel Coronavirus-Infected Pneumonia Included in Legal Infectious Disease Management. 20 January 2020. http://www.nhc.gov.cn/xcs/fkdt/202001/e4e2d5e6f01147e0a8df3f6701d49f33.shtml.

[48] National Health Commission The Party Group of the National Health and Health Commission Conveyed the Spirit of Implementing the Important Instructions of General Secretary Xi Jinping and the Instructions of Premier Li Keqiang to Study and Deploy the Pneumonia Epidemic Prevention and Control of Novel coronavirus infection. 21 January 2020. http://www.nhc.gov.cn/xcs/fkdt/202001/135b650259f647869bf7558154e84d8d.shtml.

[49] Bachman D. The Economic Impact of COVID-19 (Novel Coronavirus) COVID-19 could Affect the Global Economy in Three Main Ways. Deloitte March 03. https://www2.deloitte.com/us/en/insights/economy/covid-19/economic-impact-covid-19.html.

[50] Lampton D. (1974). The Politics of Public Health in China: 1949–1969. Ph.D. Thesis.

[51] Jingjing Y. (2019). Analysis of Health Policy Based on Government Collective Behavior in China. Med. Soc..

[52] Joseph W., William J. (2010). Ideology and Chinese Politics. Politics in China: An Introduction.

[53] Eric M. (2018). Varieties of Capitalism and Sustainable Development: Institutional Complementarity Dynamics or Radical Change in the Hierarchy of Institutions?. J. Econ. Issues.

[54] Simiao C., Zongjiu Z., Yang J., Wang J., Zhai X., Bäringhausen T., Wang C. (2020). Fangcang Shelter Hospitals: A Novel Concept for Responding to Public Health Emergencies. Lancet.

[55] Amable B., Stefano P. (2009). A Neorealist Approach to Institutional Change and the Diversity of Capitalism. Socio-Econ. Rev..

[56] Christian L. (2007). An Institutional Hierarchy to Combat the Fragmentation of International Law: Has the ILC Missed an Opportunity. N. Y. Univ. J. Int. Law Politics.

[57] Amable B. (2003). The Diversity of Modern Capitalisms.

[58] Robert B., Saillard Y. (2005). Régulation Theory: The State of the Art.

[59] Iannello K. (1992). Decisions without Hierarchy.

